# Pan-urethral stricture with epidermolysis bullosa (EB): A case report

**DOI:** 10.1016/j.eucr.2021.101855

**Published:** 2021-09-16

**Authors:** Rayka Sharifian, Saeid abouei, Shideh moftakhari hajimirzaei, Jalil Hosseini, Ali Mohammad Mirjalili

**Affiliations:** a Men's Health and Reproductive Health Research Center, Shahid Beheshti University of Medical Sciences, Tehran, Iran; b Student Research Committee, Faculty of Medicine, Shahid Sadoughi University of Medical Sciences, Yazd, Iran

**Keywords:** Pan-urethral stricture, Epidermolysis bullosa, Clean intermittent catheterization

## Abstract

EB is an inherited skin disease that presents with the symptom of skin blisters following trauma, Involvement of the Urogenital system in these people is in the form of meatal stenosis, urinary tract infection and ureteral stricture. We introduce patient with EB and urethral involvement. A 32-year-old man without prenatal problems whose EB skin symptoms started at 6 months of age and urinary symptoms started at 12 years of age. Skin changes on prenatal ultrasound at 18 and 21 weeks of gestation will help in the diagnosis. Follow-up of EB patients with ultrasound will help to identify urogenital complications earlier.

## 1 Introduction

Epidermolysis bullosa (EB) is a group of genetic disorders characterized by a particular shift to blister formation after mild trauma. According to which part of skin is involved, EB is categorized to intraepidermal, junctional, and epidermal forms. Involvement of the genitourinary system is uncommon, with past reported problems including meatal stenosis, infection, and hydronephrosis secondary to ureteral obstruction.^1^ We present person who with EB and urethral stricture.

## 2 Case presentation

**Fig. 1:**
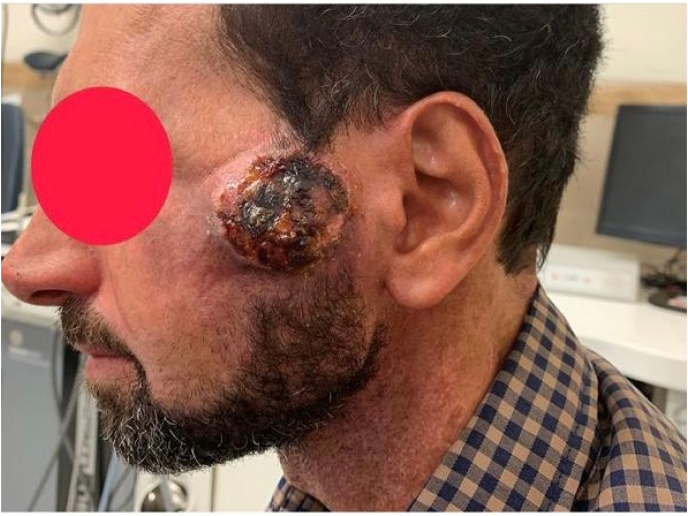
Temporal SCC.

**Fig. 2:**
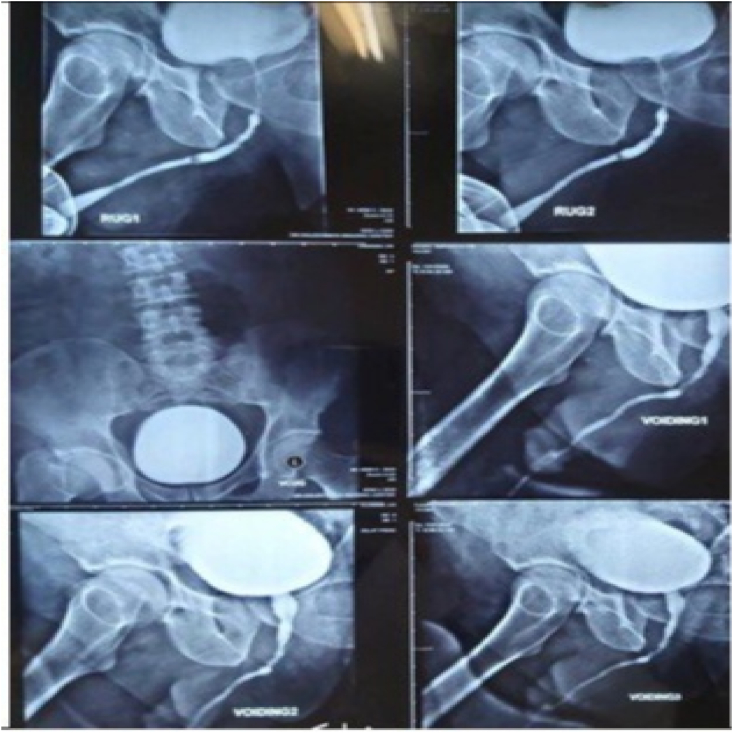
RUG and VCUG that shows Urethral stricture.

A 32-year old man was born without prenatal complications. The first episode of skin lesions has appeared since he was six-month-old and the diagnosis of EB was achieved. His mild obstructive urinary symptoms have begun twelve years ago and he has gone several cystoscopy and urethral dilation. Five years ago, the patient visited our clinic while the symptoms got worse requiring cystostomy. Another cystoscopy dilation was done initiating by 8 French to 16 French and the stricture involving from meatus till prostatic urethra was noticed. He was doing clean intermittent catheterization (CIC) every day. Physical examination revealed several bullous lesions on different parts of his body and a chronic scar on the head (left parietal side) (Fig. 1). Despite of recommendation of further investigation of it, he had sufficed with home remedies and ultimately it was diagnosed as squamous cell carcinoma. Lesion resection in conjunction with radical axillary lymphadenectomy was done for him. Laboratory data was normal. Retrograde urography (RUG) revealed several strictures in bulbar, membranous and penile urethra secondary to urethritis or post-traumatic urethral stricture (Fig. 2). Finally, it should be mentioned that his brother had also referred with EB & urogenital complications (meatal stenosis, urinary tract infection and ureteral stenosis) that underwent marsupialization (opening meatal orifice in penoscrotal region).

## 3 Discussion

In this paper, the 32-year-old man with EB disorder and complaint of urethral stricture was investigated. EB is a group of skin disorders featuring in non-inflammatory blistering of tissues including stratified epithelium caused by trauma in a wide range of severity. Skin grade engagement determines the severity of the disease.^2^ It also establishes a three-type classification of the disease along with the clinical presentation: 1- EB simplex is the most common, in association with basal layer disruption and benign lesions in hands and feet without long-term complications, 2- Junctional EB (JEB) is indicated by rupture in basement membrane without scarring with possibility of skin atrophy, and 3- Dystrophic EB (DEB) is related to derma involvement and more morbidity. The epithelium of trachea, esophagus and urinary tract can be involved. Urogenital complications were first reported by Kretkowski in 1973. It was stated that a 3 -year-old boy presenting with dysuria, urethral meatal stenosis, ulcerations of the glans, with distension of bladder and hydronephrosis.^3^ Then in 1998, Glazier and Zaontz presented a comprehensive review of the only other 14 cases had published to that date, and reported on the outcome of ureterosigmoidostomy in their patients.^2^ A detailed review of major genitourinary complications (meatal stenosis, urinary retention, bladder hypertrophy, hydronephrosis, and pyelonephritis) from the American National EB Registry was presented by Fine et al., in 2004.^4^ They also evaluated the outcomes of the performed procedures in patients with EB. They concluded that urological problems are often noticeable in patients with more severe subtypes; JEB and the recessive DEB variant Genitourinary complications of inherited epidermolysis bullosa: experience of the national epidermolysis bullosa registry.^4^

## 4 Conclusion

The precise cure for EB despite of many years of investigation is not available yet. The simplex type is autosomal dominant, and the junctional type is primarily autosomal recessive. If a parent has a child with this disorder, genetic counseling should be offered. Prenatal detection is now possible via ultrasound guided fetal skin biopsy, demonstrating characteristic skin changes between 18 and 21 weeks of gestation.^5^ Phenytoin was originally believed to be the most promising treatment but it was shown to be ineffective at reducing the number of blisters. Despite of scarcity of genitourinary tract complications in EB, annual follow–up, and routine urinalysis may afford earlier clues for genitourinary disease. Ultrasonography is a good diagnostic procedure to screen for possible genitourinary involvement. If any abnormalities are detected, appropriate imaging should be performed such as VCUG or diuretic renography, and a specialist (nephrologist and urologist) consult should be considered.
